# Rapid Visual Detection of Feline Panleukopenia Virus Using Colorimetric Loop-Mediated Isothermal Amplification Assay

**DOI:** 10.3390/vetsci13070674

**Published:** 2026-07-11

**Authors:** Shushuai Yi, Han Zhao, Wanyi Li, Yanmeng Liu, Chao Yang, Wanli Sha, Jiangting Niu, Baishuang Yin

**Affiliations:** 1 College of Animal Science and Technology, Jilin Agricultural Science and Technology University, Jilin 132101, China; yishushuai888@jlnku.edu.cn (S.Y.);; 2 Jilin Zhengye Biological Products Co., Ltd., Jilin 132101, China

**Keywords:** feline panleukopenia virus, colorimetric LAMP, visual detection, point-of-care testing, clinical application

## Abstract

Feline panleukopenia is an acute, highly contagious, and fatal infectious disease, posing a serious threat to the health and conservation of felids. It is necessary to develop diagnostic tools suitable for point-of-care testing (POCT). Herein, a colorimetric loop-mediated isothermal amplification (LAMP) assay targeting the FPV VP2 gene was developed using cresol red as a colorimetric indicator. The colorimetric LAMP enables result interpretation by visual observation of a color change from violet to yellow after amplification at 64 °C for 40 min. The limit of detection was 18.38 copies/µL for pMD-VP2 plasmid and 10^1.62^ TCID_50_/mL for crudely extracted nucleic acids. This method exhibits no cross-reactivity with other common feline pathogens, and shows good repeatability. Nevertheless, this method is unable to differentiate FPV from canine parvovirus (CPV) owing to their high genetic similarity. Testing of 153 clinical samples revealed a 40.52% (62/153) positive rate, with 98.04% agreement compared to a commercial qPCR kit (42.48% positive rate). Collectively, the developed colorimetric LAMP assay offers a specific, sensitive, simple and visual approach for FPV detection, demonstrating great potential for field point-of-care applications.

## Simple Summary

Feline panleukopenia virus (FPV) causes severe clinical manifestations and carries a potential risk of cross-species transmission, posing a significant threat to the health of multiple animal species. In this study, we developed a colorimetric LAMP assay that allows result determination by observation of color changes with the naked eye. Based on the VP2 gene of FPV, this assay achieved a 95% limit of detection (LOD) of 18.38 copies/µL for pMD-VP2 plasmid and 10^1.62^ TCID_50_/mL for crudely extracted nucleic acids, under 64 °C incubation for 40 min. The newly developed method allows for closed-tube detection and visual readout, demonstrating good specificity and high sensitivity, and yielding results nearly identical to those of commercial qPCR kits. Given its simplicity, low cost, and visual readout, this method represents a practical diagnostic tool for FPV detection under field conditions.

## 1. Introduction

Feline panleukopenia (FPL), caused by feline panleukopenia virus (FPV), is an acute, highly contagious, and highly fatal viral disease. FPV belongs to the genus *Protoparvovirus* of the family *Parvoviridae*, and shares a common ancestor with canine parvovirus type 2 (CPV-2), mink enteritis virus (MEV), and raccoon parvovirus (RPV), with high sequence similarity among these viruses. According to the latest classification criteria of the International Committee on Taxonomy of Viruses (ICTV), they are collectively categorized as *Carnivore protoparvovirus* 1. FPV is a non-enveloped virus with a diameter of approximately 20–25 nm, and has a single-stranded DNA genome about 5200 nt in length, which contains two major open reading frames (ORFs). The first ORF encodes non-structural proteins NS1 and NS2, which are pivotal for viral replication and gene regulation [1]. The second ORF encodes the capsid proteins VP1 and VP2. VP2 is the predominant protein, constituting up to 90% of the viral capsid, and is the primary determinant of antigenicity, host range, and receptor binding. VP2 is a highly immunogenic protein capable of self-assembling into virus-like particles that elicit neutralizing antibodies. FPV infection produces fever, anorexia, vomiting, hemorrhagic diarrhea, and a marked reduction in circulating white blood cells (panleukopenia) [2]. The virus targets rapidly dividing cells in the intestinal mucosa, bone marrow, and lymphoid tissues, leading to immunosuppression and high mortality, especially in unvaccinated kittens, whose mortality rates can exceed 90%. Furthermore, FPV exhibits a broad host range beyond domestic cats, including various carnivores such as dogs [3], cheetahs [4], and lions [5], which pose a serious threat to the health of domestic cats and wild felids worldwide.

Currently, colloidal gold test strips, conventional PCR, quantitative PCR (qPCR), and nanoPCR have been widely applied for the specific detection of FPV [6,7]. Colloidal gold test strips allow for rapid, user-friendly detection but generally suffer from suboptimal analytical sensitivity. PCR, qPCR, and nanoPCR offer high sensitivity and strong specificity, but they are costly, time-consuming and not suitable for resource-limited primary veterinary clinics and field settings. There is a growing urgent need for point-of-care testing (POCT) methods suitable for feline transactions, health examinations, and home diagnosis.

Loop-mediated isothermal amplification (LAMP), first reported by Notomi et al. in 2000 [8], is an isothermal amplification technique (IAT). This method employs a set of 4–6 primers that recognize 6–8 distinct regions of the target gene, coupled with a strand-displacing *Bst* DNA polymerase to enable efficient nucleic acid amplification under constant temperature conditions. LAMP amplicons can be end-point visually detected by gel electrophoresis, turbidimetric analysis, colorimetric readout, and lateral flow dipsticks, as well as can be quantitatively detected by fluorescence dyes and multiple probes [9,10]. Owing to its rapidity, operational simplicity, result visualization, high specificity, and exceptional sensitivity, LAMP has been widely applied in the clinical diagnostics of bacteria, viruses, and parasites [11].

In this study, we developed a novel colorimetric LAMP assay that uses cresol red as a pH indicator for rapid visual detection of FPV. This method is expected to provide a simple, cost-effective, specific and sensitive diagnostic solution, holding great promise for FPV infection testing in primary veterinary clinics and on-site field applications.

## 2. Materials and Methods

### 2.1. Viral Strains, Plasmid and Clinical Samples

FPV isolate CC-02/16, feline herpesvirus 1 (FHV-1) isolate CH-B, feline calicivirus (FCV) isolate CH-JL2, canine parvovirus (CPV) isolate CC-03/17, feline coronavirus (FCoV)-positive ascites, and *Escherichia coli* (*E. coli*) were identified and preserved in our laboratory. A recombinant plasmid pMD-VP2 carrying the complete VP2 gene of FPV isolate CC-02/16 was constructed and sequenced (GenBank ID: MF541121) in our previous study. A total of 153 fecal samples were collected from cats in Jilin Province, China. All fecal samples were homogenized and resuspended in 1 mL of phosphate-buffered saline (PBS), followed by centrifugation at 10,000× *g* for 10 min at room temperature. Then, the supernatants were harvested and stored at −80 ° C until nucleic acid extraction.

### 2.2. Nucleic Acid Extraction and Quality Control

Total nucleic acid was extracted from viral cultures and clinical samples using the FastPure Viral DNA/RNA Mini Kit (Vazyme, Nanjing, China) according to the manufacturer’s protocol. The concentration and purity of the extracted nucleic acid were evaluated using a NanoDrop microvolume spectrometer (Thermo Scientific, Wilmington, NC, USA). Nucleic acid was considered qualified for downstream analysis when the A260/A80 and A260/A230 ratios were between 1.8 and 2.0. Qualified samples were stored at −20 °C until further use.

### 2.3. Primers Design

The VP2 gene sequences of epidemic FPV, CPV-2 and MEV strains were retrieved from the NCBI database and subjected to multiple sequence alignment using MEGA version 7.0 software. The alignment results are presented in Table S1. Based on the conserved regions identified in the VP2 gene, three sets of LAMP primers for the specific detection of FPV were designed using Primer Explorer version 5 software (https://primerexplorer.eiken.co.jp/lampv5e/index.html (accessed on 21 June 2025)) following the NEB LAMP primer design criteria (https://www.neb.cn/zh-cn/tools-and-resources (accessed on 21 June 2025)). Each primer set comprised outer primers (F3 and B3), inner primers (FIP and BIP), and loop primers (LF and LB). The specificity, dimer formation, and hairpin structures of all primers were evaluated using the online software Primer-Blast and MFEprimer (https://m4.igenetech.com/mpref (accessed on 22 June 2025)). All primers were synthesized by Comate Bioscience Co., Ltd. (Jilin, China). The oligonucleotide sequences of all primer sets are shown in Table S2.

### 2.4. Establishment and Optimization of Colorimetric LAMP Assay

The colorimetric LAMP assay was performed using *Bst* 3.0 DNA Polymerase (New England Biolabs, Ipswich, MA, USA), with cresol red as pH-sensitive colorimetric indicator. The total reaction volume was 25 μL, and the final concentrations of all reaction components are listed in Table 1. Viral DNA from FPV isolate was used as the positive control, while nuclease-free water served as the negative control throughout all reactions. To establish and optimize this method, real-time fluorescence LAMP supplemented with 1 × EvaGreen fluorescent dye was applied for performance evaluation. The LAMP reaction was carried out at 65 °C for 45 min using either a metal bath or a LightCycler^®^ 96 ( Roche Ltd., Basel, Switzerland). For real-time fluorescence LAMP, the fluorescence signals were collected every 30 s. The results were determined by fluorescence amplification curve and color change with the naked eye. A violet color indicates a negative reaction, while a yellow color indicates a positive reaction. The LAMP amplicons were further analyzed by 2% agarose gel electrophoresis.

To achieve the optimal performance, the following parameters were systematically optimized using a one-variable-at-a-time (OVAT) method: (1) primer set; (2) reaction temperature ranging from 59 °C to 65 °C with 1 °C gradient interval; (3) inner primers concentrations; (4) loop primers concentrations; (5) MgSO_4_ concentration; (6) dNTP mixtures concentrations; (7) *Bst* 3.0 polymerase concentrations; (8) cresol red concentrations; and (9) reaction duration ranging from 10 to 60 min with 10 min gradient interval. The optimized settings of each parameter are summarized in Table 1. The optimal conditions were determined based on a comprehensive evaluation of the time-to-positivity (Tp) values, endpoint fluorescence values, visual color change in the reaction tubes, and the bands observed on agarose gel electrophoresis. To eliminate errors, all reactions were performed in triplicate.

### 2.5. Specificity Analysis

To evaluate specificity, total nucleic acid extracted from FHV-1, FCV, FCoV, CPV and *E. coli* was first assessed for quality using NanoDrop microvolume spectrometer, and subsequently tested under optimized reaction conditions. Furthermore, the cycle threshold (Ct) values of the extracted nucleic acid were determined using commercial qPCR/qRT-PCR kits (Scenk, Beijing, China). The quality assessment data and Ct values are presented in Table S3.

### 2.6. Sensitivity Analysis and Limit of Detection (LOD) Determination

The plasmid pMD-VP2 was used as a reference standard for analytical sensitivity evaluation. The plasmid was 10-fold serially diluted with nuclease-free water to generate a concentrations gradient ranging from 1 copy/μL to 10^8^ copies/μL. The colorimetric LAMP assay was applied to detect each serial dilution, with three parallel reactions set for each concentration, and the experiment was repeated three times independently. The detection range of this method was preliminarily determined based on the detection results. To calculate the 95% LOD, the plasmid was further diluted within the preliminarily determined detection range to generate five concentration gradients of 1, 5, 10, 25, 50, and 100 copies/μL. Each concentration was tested in twenty replicates. Probit regression analysis was conducted using MedCalc 23.0 software to fit the dose–response relationship between template concentration and positive detection rate, and the 95% LOD value along with its corresponding 95% confidence interval (95% CI) was derived from the fitted model. In parallel, a commercial FPV qPCR kit (Scenk, Beijing, China) was used to detect the plasmid pMD-VP2 according to the manufacturer’s instructions, and its 95% LOD was calculated as described above.

To better simulate the rapid POCT workflow for clinical samples, serial diluted cell cultures of FPV isolate CC-02/16 were mixed with the processed supernatant of FPV-negative feline fecal samples to prepare simulated clinical specimens. A total of 100 μL of each simulated sample (containing FPV at a defined titer of 10^3.75^, 10^2.75^, 10^1.75^, 10^1.05^, 10^0.75^, and 10^0.05^ TCID_50_/mL) was subjected to rapid viral DNA extraction using a universal nucleic acid release reagent (Amp-future, Changzhou, China). Using the crude nucleic acid extracts as the template, both the colorimetric LAMP assay and commercial FPV qPCR kit were employed for detection, with twenty replicates per concentration. The 95% LOD under the rapid nucleic acid extraction condition was calculated as described above.

### 2.7. Repeatability Analysis

To evaluate the repeatability, three diluted plasmids with the concentration of 10^2^ copies/μL, 10^4^ copies/μL and 10^6^ copies/μL representing strongly, moderately, and weakly positive samples, respectively, were tested using the colorimetric assay. Eight replicates were performed for each concentration to evaluate intra-assay reproducibility, and the experiment was performed independently three times to assess inter-assay reproducibility.

### 2.8. Application of the Colorimetric LAMP Assay on Clinical Samples

A total of 153 fecal samples were used to evaluate the diagnostic performance of the developed assay for detecting FPV. The detection results were compared with those of a commercial FPV qPCR kit to evaluate the coincidence rate, comparative sensitivity and specificity using a two-by-two table as previously described [12]. Kappa values were calculated using MedCalc 23.0 software.

## 3. Results

### 3.1. Validation of LAMP Primer Sets for FPV Detection

**Figure 1:**
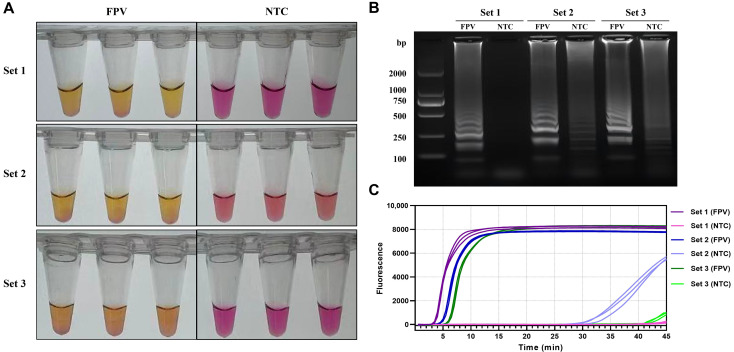
Screening results of primer sets for the colorimetric LAMP assay against FPV. (**A**) Results of visual observation: yellow color indicates positive, while violet color indicates negative. (**B**) Electrophoretic analysis results. (**C**) Results of fluorescence amplification curve.

Three primer sets targeting the FPV VP2 gene were evaluated to select the optimal set. All primer sets produced a violet-to-yellow color change with FPV DNA. Negative controls with sets 1 and 3 remained violet, whereas the negative control with set 2 turned pink (Figure 1A), likely due to a pH shift caused by primer-dimer formation. Electrophoresis analysis confirmed that only set 1 yielded bright, clear ladder-like bands in the positive reaction without bands in the negative control (Figure 1B). While sets 2 and 3 produced non-specific bands in negative controls. FPV nucleic acid contamination was excluded via repeated validation, and the observed non-specific bands were likely attributed to non-specific amplification caused by primer dimer formation. Additionally, primer set 1 showed the shortest Tp value and the highest endpoint fluorescence intensity. Non-specific amplification was detected in the negative controls with sets 2 and set 3 (Figure 1C). Based on the above results, primer set 1 was selected for the colorimetric LAMP assay development.

### 3.2. Optimization of the Colorimetric LAMP Reaction Conditions

**Figure 2:**
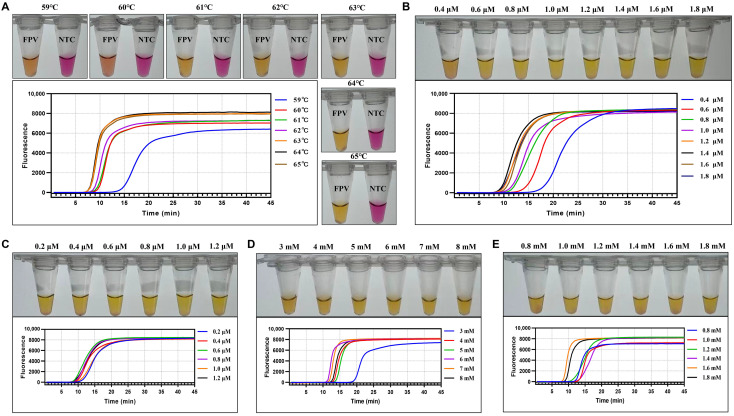
Optimization results of reaction conditions for the colorimetric LAMP assay. (**A**) Optimization of reaction temperature; (**B**) Optimization of the concentration of loop primers; (**C**) Optimization of the concentration of inner primers; (**D**) Optimization of the concentration of MgSO_4_; (**E**) Optimization of the concentration of dNTPs. Yellow color indicates positive, while violet color indicates negative.

**Figure 3:**
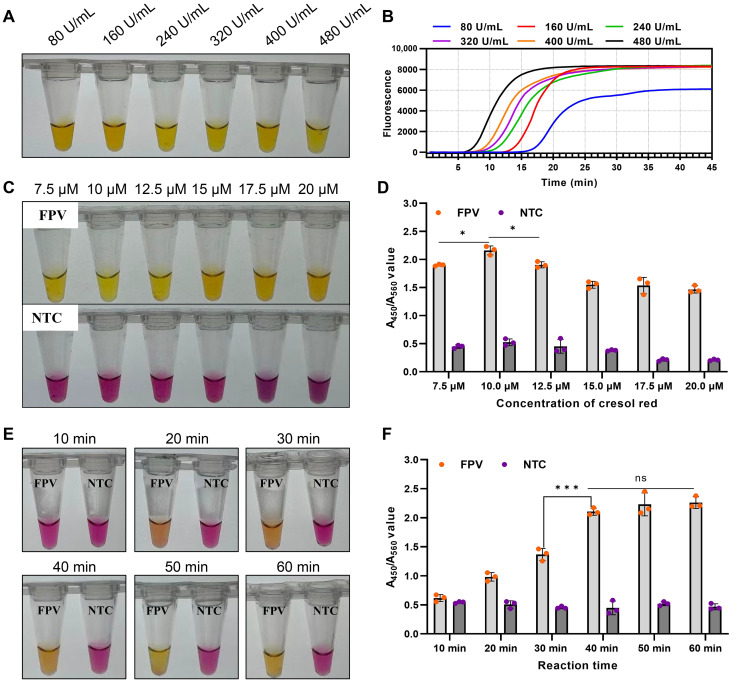
Optimization results of the concentration of *Bst* polymerase and cresol red, and reaction time. (**A**,**B**) Optimization of the concentration of *Bst* polymerase; (**C**,**D**) Optimization of the concentration of cresol red: (**C**) shows the visual assessment of color change and (**D**) shows the quantitative analysis results based on A_450_/A_560_ ratio. (**E**,**F**) Optimization of the reaction time: (**E**) shows the visual assessment of color change and (**F**) shows the quantitative analysis results based on A_450_/A_560_ ratio. Yellow color indicates positive, while violet color indicates negative. ns, no significant difference; *, *p* < 0.05; ***, *p* < 0.001.

To determine the optimal reaction conditions of the colorimetric assay, key parameters were systematically optimized as single variables. The optimal conditions were determined based on Tp value, endpoint fluorescence values, visual color change, and electrophoresis analysis. For reaction temperature optimization, this method produced a violet-to-yellow (orange) color changes in positive tubes across the range of 59–65 °C. When the reaction temperature was at 63–65 °C, the positive reaction yielded the lowest Tp, the highest endpoint fluorescence, and the clearest yellow color, with no significant differences among them (Figure 2A). Hence, 64 °C was selected as the optimal temperature for subsequent LAMP reactions. For primers concentration optimization, the lowest Tp values, highest fluorescence intensity, as well as the clearest yellow color were observed in reactions with the final concentration of inner primers (FIP/BIP) at 1.4 μM each (Figure 2B) and loop primer (LF/LB) at 0.6 μM each (Figure 2C), suggesting the optimal primer concentrations. Subsequently, the final concentrations of MgSO_4_ and dNTPs were optimized, and the results demonstrated that 6 mM (Figure 2D) and 1.6 mM (Figure 2E) yielded products with lowest Tp values and highest fluorescence intensity compared with other concentrations. Consequently, 6 mM and 1.6 mM were identified as the optimal MgSO_4_ and dNTPs concentration, respectively. The amount of *Bst* polymerase was further optimized. The reaction with 80–480 U/mL *Bst* polymerase produced a visible color change. At 80–160 U/mL, the reaction color was orange rather than bright yellow, whereas at 240–400 U/mL, the color was brighter yellow, accompanied by lower Tp values and higher fluorescence intensity, with no significant differences among these concentrations. All evaluated indicators were optimal at 480 U/mL (Figure 3A,B). Balancing amplification efficiency and cost-effectiveness, 320 U/mL was determined as the optimal final concentration of Bst DNA polymerase.

The colorimetric LAMP assay relies on the color change in the pH-sensitive indicator cresol red for visual detection. The concentration of cresol red in the reaction mixture was optimized by measuring absorbance at 560 nm (A_560_) and 450 nm (A_450_) using a micro-spectrophotometer (Thermo Scientific, Wilmington, NC, USA), with the optimal concentration determined based on the A_450_/A_560_ ratio and the observed color change. Visually, 15–20 µM cresol red produced an orange color in positive reactions, whereas 7.5–12.5 µM yielded a bright yellow, exhibiting a pronounced color contrast against the violet of negative reactions (Figure 3C). A_450_/A_560_ analysis showed that the ratios of all negative controls remained below 0.6, and the ratio of positive reactions peaked at 2.16 ± 0.08 at 10 µM, gradually declining at higher concentrations (Figure 3D). Considering both the color change and the A_450_/A_560_ ratio, 10 µM was identified as the optimal cresol red concentration.

Subsequently, to determine the minimum reaction time, 10,000-fold diluted FPV viral DNA was incubated at 64 °C for 10 to 60 min with 10 min gradient interval. A visible color change from violet to orange was first observed at 20 min, and the color developed to a clear and stable yellow after 40 min (Figure 3E). A_450_/A_560_ analysis further confirmed that the ratio reached a high level at 40 min, with no significant difference compared to 50–60 min (Figure 3F), making 40 min the optimal reaction time.

### 3.3. Detection Procedure of the Colorimetric LAMP Assay

Following systematic optimization, a colorimetric LAMP assay for FPV detection was established using the primer set listed in Table 2. Clinical samples were subjected to nucleic acid extraction, after which 5 μL of the extracted nucleic acid was added to a total volume of 20 μL reaction mixture containing 2.5 μL of 10 × LAMP mixture (200 mM Tris-HCl, 100 mM (NH_4_)_2_SO_4_, 1.5 M KCl, 60 mM MgSO_4_, 16 mM dNTPs, 1% Tween-20, pH 8.8), 2.5 μL of 10 × primers mixture (2 μM each of F3 and B3, 14 μM each of FIP and BIP, and 6 μM each of LF and LB), 1.0 μL of cresol red (250 μM), 1.0 μL of *Bst* 3.0 polymerase (8000 U/mL), and 13 μL of nuclease-free water. The amplification was performed at 64 °C for 40 min using a metal bath or a water bath. Results were interpreted by colorimetric observation with the naked eye: violet indicated a negative reaction, while yellow indicated a positive reaction.

### 3.4. Specificity of the Colorimetric LAMP Assay

**Figure 4:**
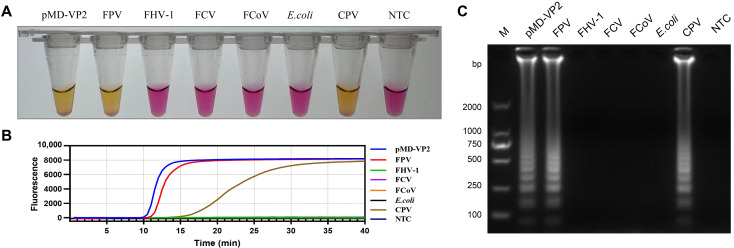
Specificity of the colorimetric LAMP assay. (**A**) Results of visual observation: yellow indicates a positive reaction, while violet indicates a negative reaction. (**B**) Fluorescence amplification curve. (**C**) Electrophoretic analysis. M, DL 2000 DNA marker; NTC, negative control.

As shown in Figure 4, reactions containing FPV DNA and plasmid pMD-VP2 displayed a violet-to-yellow color change, positive amplification curves, and characteristic ladder-like bands. In contrast, no positive reactions were observed in any of the other tested feline pathogens or the negative control. Given that FPV and CPV both belong to the species *Carnivore protoparvovirus* 1 and share over 98% nucleotide identity in the VP2 gene, they are inherently difficult to discriminate using molecular detection methods. Evaluation of this method’s specificity for CPV yielded positive amplification. Further testing with different concentrations of CPV DNA consistently produced positive results, with a detection range identical to that for FPV (Figure S2). Additionally, sequencing of the smallest band of the amplification products confirmed 100% identity to the target genes of both FPV and CPV. These results demonstrate that this method exhibits no cross-reactivity with other common feline pathogens, but it cannot distinguish between the closely related FPV and CPV.

### 3.5. Sensitivity of the Colorimetric LAMP and Comparison with qPCR

**Figure 5:**
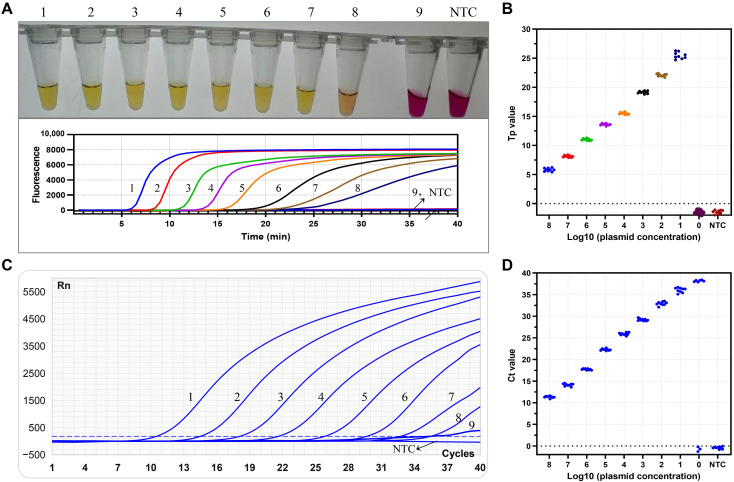
Comparative sensitivity of the colorimetric LAMP assay and qPCR. (**A**,**C**) Sensitivity analysis of the colorimetric LAMP (**A**) and qPCR (**C**). 1–9: 10^8^-1 copies/μL of plasmid concentration, respectively; NTC: negative control. (**B**,**D**) Statistical analysis of detection results for the colorimetric LAMP (**B**) and qPCR (**D**) with nine replicates at different plasmid concentrations. Yellow color indicates positive, while violet color indicates negative.

To determine the sensitivity of the colorimetric LAMP assay, 10-fold serial dilutions of the pMD-VP2 plasmid, with concentrations ranging from 1 copy/μL to 10^8^ copies/μL, were tested, and the performance was compared with that of a commercial qPCR kit. Each dilution was replicated a total of nine times. The results showed that the colorimetric LAMP assay produced a distinct color change from violet to yellow concentrations ranging from 10 to 10^8^ copies/µL, while all replicates at 1 copy/µL remained negative (Figure 5A,B). In contrast, the commercial qPCR assay was able to detect at concentrations from 1 to 10^8^ copies/µL; however, at 1 copy/µL, 3 out of 9 replicates yielded negative results (Figure 5C,D).

**Figure 6:**
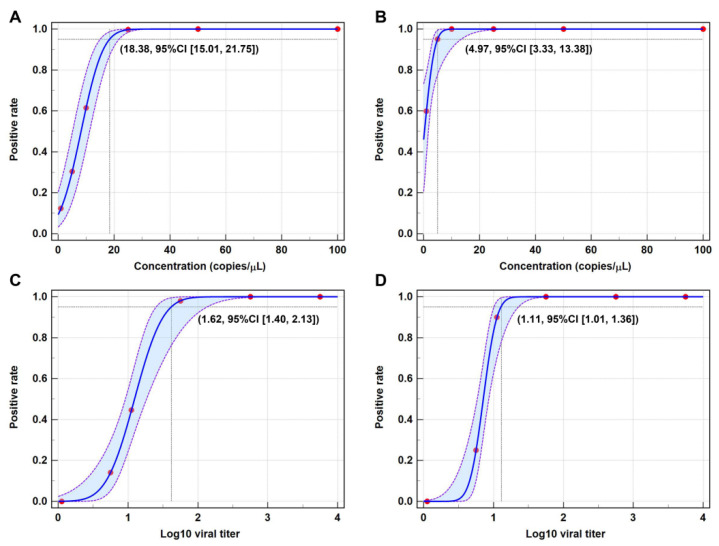
Limit of detection (LOD) and confidence interval analysis for colorimetric LAMP and qPCR. (**A**) Probit regression analysis for detecting pMD-VP2 plasmid using colorimetric LAMP. (**B**) Probit regression analysis for detecting pMD-VP2 plasmid using qPCR. (**C**) Probit regression analysis for detecting crudely extracted nucleic acid from simulated samples using colorimetric LAMP. (**D**) Probit regression analysis for detecting crudely extracted nucleic acid from simulated samples using qPCR. The blue solid line represents the probit regression curve of the detection probability at each plasmid concentration based on 20 replicate tests, and the red solid circles indicate the observed positive rates of the replicate detections. The purple dashed lines represent the 95% CI. The 95% LOD with 95% CI of the assay is marked in the figure.

To further determine the LOD at the 95%CI for both two assays, 20 replicate detections were performed using the pMD-VP2 plasmid at six different concentrations (1, 5, 10, 25, 50, and 100 copies/μL). Based on probit regression analysis, the 95%LOD of the colorimetric LAMP for the pMD-VP2 plasmid was 18.38 copies/µL, with a 95%CI of 15.01–21.75 copies/µL (Figure 6A). In comparison, the 95%LOD of the qPCR for the pMD-VP2 plasmid was 4.97 copies/µL, with a 95%CI of 3.33–13.38 copies/µL (Figure 6B). Furthermore, crudely extracted nucleic acids were obtained from simulated clinical samples (containing 10^3.75^, 10^2.75^, 10^1.75^, 10^1.05^, 10^0.75^, and 10^0.05^ TCID_50_/mL FPV cultures, respectively) and tested by both colorimetric LAMP and qPCR to evaluate the sensitivity and LOD of the two methods for POCT applications. For colorimetric LAMP, the LOD was 10^1.62^ TCID_50_/mL with 95% CI of 10^1.40^–10^2.13^ TCID_50_/mL for detecting crudely extracted nucleic acids (Figure 6C). In contrast, the LOD of the qPCR was determined to be 10^1.11^ TCID_50_/mL with 95% CI of 10^1.01^–10^1.36^ TCID_50_/mL (Figure 6D).

For the detection of both plasmid DNA and crudely extracted nucleic acids, the colorimetric LAMP assay exhibited a higher LOD compared with the commercial qPCR kit. Although its analytical sensitivity is inferior to that of qPCR, the colorimetric LAMP still serves as a robust and practical method for rapid on-site detection, combining sufficient sensitivity, visual readout, and minimal instrumentation requirements.

### 3.6. Repeatability of the Colorimetric LAMP Assay

**Figure 7:**
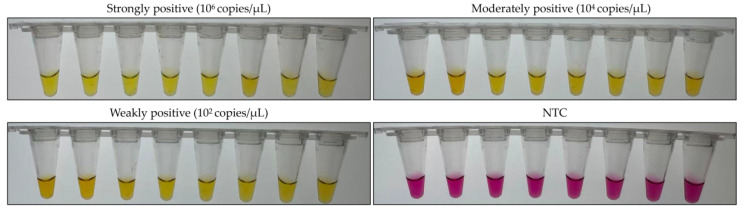
Repeatability analysis of the colorimetric LAMP assay. Each concentration was replicated eight times; yellow indicates a positive reaction, while violet indicates a negative reaction.

Plasmid pMD-VP2 with concentrations of 10^2^, 10^4^ and 10^6^ copies/μL were used as templates to evaluate the repeatability of colorimetric LAMP assay. For each plasmid concentration, all replicates in both intra-assay and inter-assay tests exhibited positive reactions with a distinct violet-to-yellow color change (Figure 7). The repeatability was further evaluated based on the coefficient of variation (CV) of the Tp values obtained from the fluorescent LAMP. As shown in Table 3, for strongly, moderately and weakly positive templates, the CVs of intra-assay and inter-assay tests were below 2% and 5%, respectively. These results indicate that this method exhibits good repeatability.

### 3.7. Evaluation of the Colorimetric LAMP on Clinical Samples

A total of 153 clinical samples were collected and tested using the developed method, and the detailed information and detection results are presented in Table S4. Sixty-two were identified as positive by this method, and all these results were fully confirmed by a commercial qPCR kit. Conversely, three samples that tested positive by the qPCR kit, all with Ct values exceeding 35.0, yielded negative results by the colorimetric LAMP assay. These three samples were retested three times, and the qPCR amplification products were purified by electrophoresis, sequenced, and subjected to sequence alignment, which further confirmed them as FPV-positive samples. Using the qPCR kit as the reference method, this method showed a comparative specificity of 100% with 95% CI of 94.79–100%, and a comparative sensitivity of 95.39% with 95% CI of 86.24–98.80%, respectively (Table 4). The agreement was 98.04%, with a kappa coefficient (k) value of 0.96, suggesting almost perfect concordance between the two methods.

## 4. Discussion

Feline panleukopenia is a highly contagious and often fatal viral disease characterized by hemorrhagic enteritis, severe diarrhea, and immunosuppression, with 50–90% mortality in unvaccinated kittens. Although widespread vaccination has effectively reduced its prevalence, outbreaks continue to occur in animal shelters, stray cats, and among incompletely vaccinated domestic cats. FPV can also infect other wild felids, including cheetah [4], lion [5], ocelot [13], and Siberian tiger [14], etc., posing a significant threat to both the health of domestic cats and the conservation of wild felids. In recent years, FPV has shown a continuously expanding host range via cross-species transmission, with infections confirmed in multiple non-feline species, including dogs [3,15], minks [16], giant pandas [17,18], Marsican brown bear [19], and monkeys [20]. Therefore, rapid, sensitive, and specific detection methods for FPV are urgently needed to enable early diagnosis, facilitate surveillance in wild carnivores, and track cross-species transmission.

LAMP, as an early-developed IAT, has been widely applied for pathogen detection. In this study, we developed a colorimetric LAMP assay using cresol red as a pH indicator for the rapid visual detection of FPV targeting the VP2 gene after primer screening and reaction condition optimization. This method enables visual detection based on the color change in cresol red (from violet to yellow), which is added prior to the reaction. The total reaction time is 40 min, and the 95% LOD is 18.38 copies/µL (95% CI: 15.01–21.75 copies/µL). In previous studies, LAMP has been applied to the detection of FPV and CPV. Morovvati et al. developed a LAMP for CPV detection, which also required 60 min and used the green fluorescence generated by post-reaction addition of fluorescent calcein for result readout, necessitating a UV lamp, with an LOD of 25 copies/µL [21]. Liu et al. reported a closed-tube SEA assay for FPV using a pH-sensitive indicator, achieving a detection time of 40 min and a LOD of 6.6 pg/µL [22]. Compared to these earlier methods, this assay employed cresol red, a pH-sensitive indicator with a color transition range of pH 7.2–8.8, which is more sensitive to pH changes than neutral red (pH 6.4–8.0). Unlike fluorescent dyes or calcein, it does not require UV or blue light excitation for visualization. Additionally, cresol red is added to the reaction mixture before amplification, enabling closed-tube detection and minimizing the risk of aerosol contamination. In terms of detection time, the incorporation of two loop primers accelerated the reaction, allowing detection within 40 min, which is superior to previously reported LAMP assays. The 95% LOD of this method is higher than the LAMP method established by Morovvati et al. [21].

Furthermore, we evaluated the LOD of the developed assay using simulated clinical samples with crudely extracted nucleic acids. The 95% LOD was determined to be 10^1.62^ TCID_50_/mL, which is lower than that of the CPV LAMP-LFD method (10^−1^ TCID_50_/mL) established by Sun et al. [23] and also inferior to that of the commercial kit used in this study. However, based on the Ct values obtained from qPCR, this method successfully detected all crudely extracted nucleic acids with Ct values < 35.0, indicating its suitability for rapid detection of clinical samples. These results also further demonstrate that commercial nucleic acid release reagent can be effectively used for the rapid extraction of FPV DNA from clinical samples, and when combined with this method, are better suited for POCT diagnosis. Overall, this method offers several advantages, including closed-tube detection, direct visual observation without excitation light source, and a more easily interpretable color transition from violet to yellow. Combined with a short detection time and considerable sensitivity, it is particularly well suited for the rapid visual detection of FPV.

Specificity analysis demonstrated no cross-reactivity with other common feline viruses. However, the detection range of this method was nearly identical for different concentrations of CPV and FPV nucleic acids, and it was unable to distinguish between the two viruses. Both FPV and CPV belong to the species *Carnivore protoparvovirus* 1 and share over 98% nucleotide identity in the VP2 gene, making them difficult to distinguish using conventional molecular assays [24]. The developed colorimetric LAMP assay used six primers targeting eight distinct regions of the VP2 gene, which limits its ability to differentiate single-nucleotide polymorphisms (SNP), making it unable to distinguish FPV and CPV. Based on the genome alignment results of circulating FPV and CPV strains, it is a challenge to identify suitable target genes for designing primers that can discriminate between FPV and CPV, a limitation that has also been corroborated by previously established LAMP, RPA, and qPCR assays [25,26]. To date, CPV infection in cats has been reported in many countries. Both FPV and CPV infections can cause clinical signs such as diarrhea and leukopenia. Although the two viruses share a high degree of genetic homology, strain-specific differences remain relevant for vaccination and antibody therapy. Therefore, differential diagnosis is crucial for developing accurate prevention and treatment strategies. Unfortunately, the current method cannot distinguish between FPV and CPV, and positive results must be confirmed and differentiated by other approaches, such as sequencing, PCR-RFLP, and qPCR-HRM [27,28]. The combination of CRISPR/Cas12a, cleavable probes, or MGB probes with LAMP has the potential to achieve SNP-specific detection [11,29]. Whether these approaches can be applied to the differential diagnosis of FPV and CPV will be a key focus of our future research.

Currently, isothermal amplification technologies and qPCR have been widely applied in the clinical diagnosis of FPV. We have summarized the relevant detection methods developed in recent years, as presented in Table S5. Recombinase polymerase amplification-lateral flow strip (RPA-LFS) achieves a detection time of 15–25 min, with results that can be visually observed via the LFS, and offers a LOD of 10–100 copies/µL [30,31]. The integration of CRISPR/Cas12a with RPA substantially enhances sensitivity, achieving a LOD of 1–5 copies/µL, albeit at the cost of a longer detection time [32,33,34]. Although RPA features a short detection time and high sensitivity, it requires multiple enzymes, which increases the overall detection cost. qPCR provides stable results and enable multiplex detection of multiple pathogens [7,28,35,36]; however, they are limited by long detection times and the need for complex equipment. Compared with these methods, the developed colorimetric LAMP assay offers distinct advantages, including a moderate detection time, higher cost-effectiveness, considerable sensitivity, and no requirement for complex equipment.

We further evaluated the clinical performance of the developed colorimetric LAMP by testing 153 clinical samples. Compared with a commercial qPCR kit, this method achieved an analytical specificity of 100%, with a lower 95% CI bound of 94.79%, indicating an excellent ability to exclude negative results. The analytical sensitivity was 95.39% (95% CI: 86.24–98.80%). The relatively wide confidence interval is primarily attributable to the limited sample size in this study, which introduced considerable sampling error in the sensitivity estimate. Moreover, the lower CI bound of 86.24% suggests a certain risk of false-negative results with this method. We further conducted a stratified sensitivity analysis based on the Ct values obtained by qPCR (Table S6). The results showed that the sensitivity for strongly positive samples (Ct ≤ 25.0) and moderately (25.0 < Ct ≤ 30.0) positive samples was 100%, whereas the sensitivity for weakly (Ct ≥ 30.0) positive samples decreased to 82.35%. On the one hand, evaluation of a larger number of weakly positive samples is needed to more reliably assess the performance of this method. On the other hand, for samples with a high clinical suspicion but a negative LAMP result, confirmation using alternative diagnostic methods is recommended.

This method also has several limitations in clinical application. Nucleic acid extraction remains a major obstacle to the point-of-care deployment of LAMP assays. Although the use of nucleic acid release reagent can simplify the extraction process, and crudely extracted nucleic acids can be used for detection with this method, the development of a one-step colorimetric LAMP assay that eliminates the need for nucleic acid extraction altogether would hold greater value for clinical translation. Additionally, this method relies on color change for result interpretation, which is readily achievable for strongly and moderately positive samples; however, the color change in weakly positive samples is often inconspicuous and can easily lead to misjudgment. Establishing quantitative colorimetric detection standards for colorimetric LAMP by integrating smartphones, artificial intelligence-based image analysis, and color analysis software is also a main direction for our future development.

## 5. Conclusions

In conclusion, a colorimetric LAMP for the rapid visual detection of FPV was successfully established in this study. This method allows result determination by visual observation with a color change from violet to yellow. The developed colorimetric LAMP assay exhibited high specificity and sensitivity, and is simple, cost-effective, and time-saving, making it well suited for clinical diagnosis and epidemiological surveillance of FPV. This study provides a powerful point-of-care testing tool for the detection of FPV in field settings.

## Figures and Tables

**Table 1:** Reaction system and optimized parameters for colorimetric LAMP assay.

Parameters	Final Concentration	Optimized Settings
10 × isothermal amplification buffer (200 mM Tris-HCl, 100 mM (NH_4_)_2_SO_4_,1.5 M KCl, 20 mM MgSO_4_, 1%Tween-20, pH 8.8)	1× (contains 2 mM MgSO_4_)	1× (contains 2 mM MgSO_4_)
MgSO_4_ (100 mM)	6 mM	1–6 mM with 1 mM gradient interval (total 3–8 mM with 1 mM gradient interval)
dNTP mixture (10 mM)	1.4 mM	0.8–1.8 mM with 0.2 mM gradient interval
F3/B3 primers (100 μM)	0.2 μM	0.2 μM
FIP/BIP primers (100 μM)	1.6 μM	0.4–1.8 μM with 0.2 μM gradient interval
LF/LR primers (100 μM)	0.4 μM	0.2–1.2 μM with 0.2 μM gradient interval
Cresol red (250 μM)	10 μM	7.5–20 μM with 2.5 μM gradient interval
EvaGreen dye (50×)	1×	1×
*Bst* 3.0 polymerase (8000 U/mL)	320 U/mL	80–480 U/mL with 80 U/mL gradient interval
Template DNA	5 μL	5 μL
Nuclease-free water	To 25 μL	To 25 μL
Total reaction volume	25 μL	25 μL

**Table 2:** Oligonucleotide sequences of primers used for colorimetric LAMP assay in this study.

Primer Name	Primer Sequences (5′-3′)
FPV-F3	CCATGGAGATATTATTTTCAATGG
FPV-B3	TTGTTTGCCATGTATGTGTT
FPV-FIP	AACATCATCTGGATCTGTACCATGAACATTAATACCATCTCATACTGGAA
FPV-BIP	TTCTGTGCCAGTACACTTACTAAGAGTCTACATGGTTTGCAATCA
FPV-LF	GATATACATTTGTTGGTGTGCCACTAG
FPV-LB	ACAGGTGATGAATTTGCTACAGGAA

**Table 3:** Intra-assay and inter-assay repeatability for the colorimetric LAMP assay based on fluorescence amplification curve.

Template	Intra-Assay (Tp Values)			Inter-Assay (Tp Values)		
	Mean	SD	CV (%)	Mean	SD	CV (%)
Strongly positive (10^6^ copies/μL)	11.01	0.18	1.63	11.34	0.33	2.91
Moderately positive (10^4^ copies/μL)	15.53	0.17	1.09	15.55	0.40	2.57
Weakly positive (10^2^ copies/μL)	22.18	0.25	1.13	23.06	0.72	3.12

**Table 4:** Comparative analysis of the colorimetric LAMP assay and a commercial FPV qPCR kit for the detection of FPV in clinical fecal samples.

Colorimetric LAMP	Commercial qPCR Kit			Specificity(%) (95% CI)	Sensitivity(%) (95% CI)	*k* Value	Agreement (%)
	Positive	Negative	Total				
Positive	62	0	62	100 (94.79–100)	95.39 (86.24–98.80)	0.96	98.04
Negative	3	88	91				
Total	65	88	153				

## Data Availability

The original contributions presented in this study are included in the article/Supplementary Material. Further inquiries can be directed to the corresponding authors.

## Supplementary Materials

The following supporting information can be downloaded at https://www.mdpi.com/article/10.3390/vetsci13070674/s1, Figure S1: Electrophoretic analysis of the optimization results of reaction conditions for the colorimetric LAMP assay; Figure S2: Results of cross-reactivity testing between the colorimetric LAMP assay and different concentrations of CPV DNA; Figure S3: Electrophoretic analysis of the products from sensitivity test of the colorimetric LAMP assay; Table S1: Nucelotide sequences alignment results based on VP2 gene of circulated FPV strains; Table S2: Oligonucleotide sequences of LAMP primer sets designed in this study; Table S3: Sample information and nucleic acid assessment data of viral and bacterial strains for specificity assay; Table S4: Basic information and testing results of 153 clinical fecal samples; Table S5: Comparison of isothermal amplification techniques and quantitative PCR for the detection of FPV; Table S6: Stratified sensitivity analysis based on Ct values of qPCR.

## Abbreviations

The following abbreviations are used in this manuscript:

CPV	Canine parvovirus
Ct	Cycle threshold
FPV	Feline panleukopenia virus
FHV-1	Feline herpesvirus 1
FCV	Feline calicivirus
FCoV	Feline coronavirus
IAT	Isothermal amplification techniques
LAMP	Loop-mediated isothermal amplification
LOD	Limit of detection
ORF	Open reading frame
PCR	Polymerase chain reaction
POCT	Point-of-care testing
qPCR	Quantitative PCR
Tp	Time to positivity
