# Supplementary material for: Rapid Visual Detection of Feline Panleukopenia Virus Using Colorimetric Loop-Mediated Isothermal Amplification Assay
Source: Vet Sci. 2026 Jul 11;13(7):674. doi: 10.3390/vetsci13070674 (PMC13431338; doi:10.3390/vetsci13070674)
Supplement: Supplementary file 1 [file vetsci-13-00674-s001.zip › Supplementary Figures.pdf]

Article

Rapid Visual Detection of Feline Panleukopenia Virus Using Colorimetric Loop-mediated Isothermal Amplification Assay

Shushuai Yi [1,2,†], Han Zhao [1,†], Wanyi Li [1], Yanmeng Liu [1], Chao Yang [2], Wanli Sha [1], Jiangting Niu [1,2,*] and Baishuang Yin [1,*]

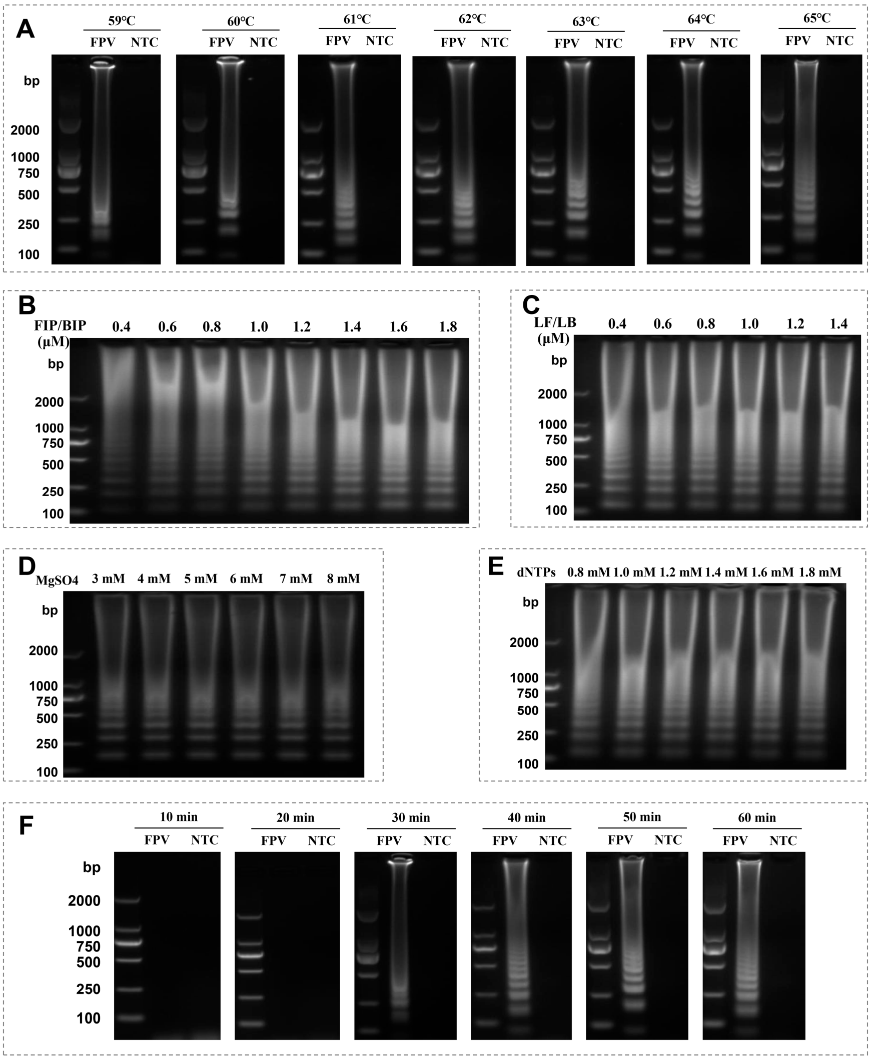

Figure S1. Electrophoretic analysis of the optimization results of reaction conditions for the colorimetric LAMP assay. (A) Optimization of reaction temperature; (B) Optimization of the concentration of loop primers; (C) Optimization of the concentration of inner primers; (D) Optimization of the concentration of $MgSO_4$; (E) Optimization of the concentration of dNTPs; (F) Determination of reaction time.

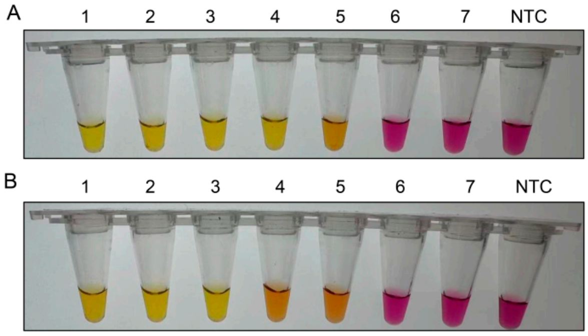

Figure S2. Results of cross-reactivity testing between the colorimetric LAMP assay and different concentrations of CPV DNA. (A) Detection results for FPV strain CC-02/16. Concentrations of samples 1–7: 45.18 ng/μL, 4.518 ng/μL, 0.4518 ng/μL, 45.18 pg/μL, 4.518 pg/μL, 0.4518 pg/μL, and 45.18 fg/μL; NTC, negative control. (B) Detection results for CPV strain CC-03/17. Concentrations of samples 1–7: 37.62 ng/μL, 3.762 ng/μL, 0.3762 ng/μL, 36.72 pg/μL, 3.672 pg/μL, 0.3672 pg/μL, and 36.72 fg/μL; NTC, negative control.

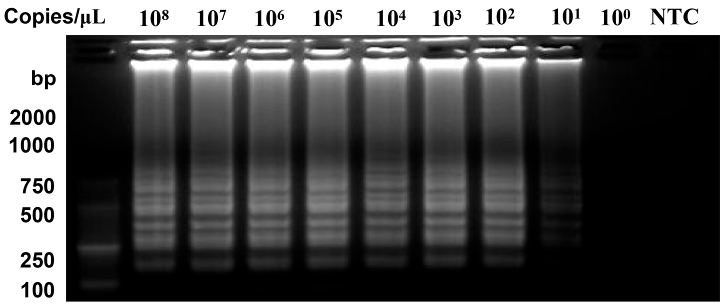

Figure S3. Electrophoretic analysis of the products from sensitivity test of the colorimetric LAMP assay.
