# Supplementary material for: Rapid Visual Detection of Feline Panleukopenia Virus Using Colorimetric Loop-Mediated Isothermal Amplification Assay
Source: Vet Sci. 2026 Jul 11;13(7):674. doi: 10.3390/vetsci13070674 (PMC13431338; doi:10.3390/vetsci13070674)
Supplement: Supplementary file 1 [file vetsci-13-00674-s001.zip › Supplementary Tables (S2, S3, S5 and S6).pdf]

Article

Rapid Visual Detection of Feline Panleukopenia Virus Using Colorimetric Loop-mediated Isothermal Amplification Assay

Shushuai Yi [1,2,†], Han Zhao [1,†], Wanyi Li [1], Yanmeng Liu [1], Chao Yang [2], Wanli Sha [1], Jiangting Niu [1,2,*] and Baishuang Yin [1,*]

Table S2. Oligonucleotide sequences of LAMP primer sets designed in this study.

Primer set	Primer name	Primer sequences (5′-3′)	Position[1]
Set 1	FPV-F3-1	CCATGGAGATATTATTTTCAATGG	3405-3428
	FPV-B3-1	TTGTTTGCCATGTATGTGTT	3611-3630
	FPV-FIP-1	AACATCATCTGGATCTGTACCAT-	3487-3509
	(1F1c-1F2)	GAACATTAATACCATCTCATACTGGAA	3433-3459
	FPV-BIP-1	TTCTGTGCCAGTACACTTACTAAGA-	3530-3554
	(1B1c-1B2)	GTCTACATGGTTTGCAATCA	3590-3609
	FPV-LF-1	GATATACATTTGTTGGTGTGCCACTAG	3460-3486
	FPV-LB-1	ACAGGTGATGAATTTGCTACAGGAA	3555-3579
Set 2	FPV-F3-2	TGAGACCAGCTGAGGTTG	3778-3795
	FPV-B3-2	GGTGTTTCTCCTGTTGTAGT	3951-3970
	FPV-FIP-2	TCCTGCTGCAATAGGTGTTTTAA-	3844-3866
	(2F1c-2F2)	ATAGTGCACCATATTATTCTTTTGAAG	3799-3825
	FPV-BIP-2	GGAGCGCAAACAGATGAAAATC-	3873-3894
	(2B1c-2B2)	TTTTTGACCATGTTGTCTACC	3927-3947
	FPV-LF-2	ATGGCCCTTGTGTAGACG	3826-3843
	FPV-LB-2	AAGCAGCAGATGGTGATCCAA	3895-3915
Set 3	FPV-F3-3	ACTCAAATGGGAAATACAGACT	3735-3756
	FPV-B3-3	GGTGTTTCTCCTGTTGTAGT	3951-3970
	FPV-FIP-3	CCTTGTGTAGACGCTTCAAAAGAAT-	3814-3838
	(3F1c-3F2)	CTATTATGAGACCAGCTGAGG	3772-3792
	FPV-BIP-3	GGAGCGCAAACAGATGAAAATCA-	3873-3895
	(3B1c-3B2)	GTTTTTTGACCATGTTGTCT	3930-3949
	FPV-LF-3	AATATGGTGCACTATAACCA	3794-3813
	FPV-LB-3	AGCAGCAGATGGTGATCCAA	3896-3915

[1]Position numbers are determined based on the complete genome sequences of FPV strain Cu-4 (GenBank ID: M38246).

Table S3. Sample information and nucleic acid assessment data of viral and bacterial strains for specificity assay

Virus/Bacteria/ Plasmid	Strains	Type	Virus titer	Nucleic acid assessment data			
				A260/A280	A260/A230	Concentration	Ct value
pMD-VP2	/	Plasmid	/	1.94	1.98	173.91 ng/μL	10.51
FPV	CC-02/16	Cell culture	$10^{5.75}$ $TCID_{50}$/mL	1.93	1.96	45.18 ng/μL	17.35
FHV-1	CH-B	Cell culture	$10^{5.50}$ $TCID_{50}$/mL	1.87	1.91	40.09 ng/μL	18.22
FCV	CH-JL2	Cell culture	$10^{5.25}$ $TCID_{50}$/mL	1.90	1.94	31.56 ng/μL	19.33
CPV	CC-03/17	Cell culture	$10^{5.38}$ $TCID_{50}$/mL	1.97	1.99	37.62 ng/μL	20.14
FCoV	/	Positive samples	/	2.01	1.92	26.35 ng/μL	24.47
E.coli	/	Positive samples	/	1.85	1.88	102.84 ng/μL	22.06

Table S5. Comparison of isothermal amplification techniques and quantitative PCR for the detection of FPV

Methods	Target gene	Reaction temperature & detection time	LOD	Instruments	Results determination	Reference
LAMP	VP2	95℃, 5 min; 60℃, 60 min	1.25 ng/μL	Metal bath	After the reaction, SYBR Green was added, and a distinct color change from orange to apple green was observed.	[25]
SEA	VP2	61℃, 40 min	6.6 pg/μL	Metal bath	Color change by the pre-addition of a pH-sensitive dye.	[22]
RPA-LFD	VP2	38℃, 15 min	100 copies/μL	Metal bath	LFS	[30]
RPA-LFDA	VP2	39℃, 25 min	10 copies/μL	Metal bath	LFS	[31]
RAA-CRISPR/Cas12a-LFS	VP2	37℃, 65 min	2.1 copies/μL	Metal bath	LFS	[32]
Two/One-tube RAA-CRISPR/Cas12a	VP2	38℃, 30 min; 37℃, 10-30 min	4.277 copies/μL	Metal bath	Fluorescent observation under blue light	[33]
One-tube RAA-CRISPR/Cas12a-LFS	VP2	38℃, 30 min; 37℃, 20 min	42.77 copies/μL	Metal bath	LFS	[33]
RPA-Cas12a-Based Fluorescence Assay	NS1	37℃, 40 min	1 copy/μL	Metal bath	Fluorescent observation under UV light	[34]
HRM-qPCR	VP2	qRT-PCR protocol 60-80 min	4.2 copies/μL	qPCR instrument	Melting curves analysis	[28]
MGB-probe qPCR	VP2	qRT-PCR protocol 60-80 min	100 copies/μL	qPCR instrument	Fluorescence amplification curves (Ct value)	[35]
TaqMan qPCR	VP2	qRT-PCR protocol 60-80 min	50 copies/μL	qPCR instrument	Fluorescence amplification curves (Ct value)	[36]
TaqMan qPCR	NS1	qPCR protocol 60-80 min	100 copies/μL	qPCR instrument	Fluorescence amplification curves (Ct value)	[37]

Table S6. Stratified sensitivity analysis based on Ct values of qPCR.

**Ct value**	**Positive for qPCR**	**Positive for LAMP**	**Sensitivity**	**95% confidence interval (CI)**
Ct≤25.0	24	24	100%	85.75%-100%
25.0＜Ct≤30.	24	24	100%	85.75%-100%
Ct≥30.0	14	17	82.35%	56.57%-96.20%
